# Event and Comment

**Published:** 1906-06-15

**Authors:** 


THE
### DENTAL REGISTER.

Vol. LX. June 15, 1906. No. 6.

### EVENT AND COMMENT.

### Prophylaxis.

We recently had the privilege of inspecting the work of the chief of the prophylaxis propoganda under conditions that are rarely possible in connection with any form of clinical demonstration, and which was not only a great pleasure but a most convincing argument for this form of treatment. By the special courtesy of Dr. D. D. Smith, of Philadelphia, some twenty dentists from the different parts of the country made a visit to Philadelphia, and inspected the mouths of over thirty patients who had received the treatment at Dr. Smith's hands, covering periods of from several months to six years. We were prepared to see good results, but were greatly surprised to see such a uniform lot of perfectly healthy and clean mouths, most of which had been redeemed from what had evidently been desperate cases of pyorrhea. We have sometimes been tempted to conclude that pyorrhea alveolaris was one of the diseases that would have to be classed as incurable. But since seeing these results, we have taken new courage and will henceforth not dispair of even the desperate conditions which sometimes come to us.

We are of the opinion that the profession generally has not given enough attention to Dr. Smith's arduous efforts to convince us that we have in prophylaxis as he interprets and practices it, one of the greatest boons to humanity that modern therapeutics has furnished us, because of its far-reaching influence on the general health of our patients. To see mouths in which the masticating functions have been almost wholly lost, with the most threatening systemic complications resulting, restored to comfortable and thorough functional condition and systemic health following as the direct result of this local treatment without any other systemic treatment, is enough to make one stop and consider whether the profession is not criminally neglecting its avowed object, in either wholly neglecting a disease which is bound, in a short time, to cause the loss of the teeth, and which occasions much sufferings and body ill health during its progress, while most of us are endeavoring to cure or prevent it by means which are more often injurious than helpful.

Dr. Smith has labored to demonstrate to the profession the value of this treatment, and has endeavored by the usual means to compel the profession to accept his statement as to its value. The indifference with which the profession has taken to this work would long since have discouraged a less determined man, but he has kept at it until he is now in a position to show actual results after a sufficient long term of years to justify the faith that has kept him so conscientiously at work during all these years. Many men, it is true, have taken up the work and think they are practicing it, either themselves or by an office assistant, or the so-called "trained nurse." We feel perfectly free to say to all such that we don't believe any ordinary assistant, and especially any however *well*-trained nurse, can have any conception of what is required in the treatment of most of the cases exhibited by Dr. Smith, much less would they have the ability to administer the proper treatment. Such treatment requires a high order of skill in not only the mere technical details of scaling and polishing the teeth, but also to secure adequate functional and esthetic results,exceptional attainments in crown and bridge work and operative dentistry, such as come only after long experience and matured judgment.

It may be that the excessive exactions of the treatment will deter many excellent dentists, who conscientiously want to do the best thing possible for their patients from taking up this work, and we sincerely trust that no one will undertake it who does not take the pains to thoroughly qualify himself for the work, or at least to get a proper conception of what it demands, and then makes an earnest effort to qualify for its practice. Indeed we are not sure, but it ought to be done as a specialty. It is in itself exacting enough to claim one's time and thought freed from the other ordinary entanglements of general practice. When prophylaxis has demonstrated its pre-eminence as a treatment, as it seems destined to do, it will undoubtedly attract more thoughtful consideration by the profession, and inevitably we shall have practitioners specializing it as they now do in oral surgery and orthodontia. The field of its application is practically unlimited, as decay of the teeth is practically universal, and deposits on the teeth and diseases of the gums are almost as common. It does not seem unreasonable to prognosticate that plate dentistry may become one of the lost arts in the next century of dentistry. Orthodontia and prophylaxis supplementing operative restorations ought to redeem the people dentally and make for longer and more comfortable living. We write this after a year of experience with prophylaxis, and after mature thought and an exceptional opportunity to view the results of Dr. Smith's work for the past six years, and we believe we are making essentially a most conservative statement.

**Prof. W. D. Miller.**

As some of our readers may know from news-paper reports, Professor Miller will return to America, and has been called to the chair of Dental Histology and Pathology in the University of Michigan, and to be the Dean of the Dental Department. This will be welcome news to his friends here and the friends of the University of Michigan. It is, however, a more significant matter for the entire profession. Dr. Miller, by his scientific researches has placed himself in a position to command the confidence of scientific workers in the dental profession the world over, and he probably has the capacity for doing and directing scientific work in his special field possessed by few other men in our profession. The Regents of the University of Michigan realize this fact and are preparing to make available the University resources and Professor Miller's knowledge and skill for the advancement of the scientific aspects of dentistry, not for the glory of their institution, but that the science of dentistry may at least keep pace with the immense strides being made in its technical or art features. The intention is to place under Professor Miller's direction the scientific education of such students as exhibit a capacity for such work, or such as promise to develope a capacity, and to establish a laboratory for research on dental subjects for all workers who may not have proper facilities in the way of instructors or equipment. It is hoped that this work shall so commend itself to the profession in general, that students having a scientific desire and qualifications may be assisted in preparing themselves for teachers and scientific workers.

There is undoubtedly great need for such a training school and it should receive the cordial support of the entire profession and we confidently expect it will.

